# Sexual Differences in Internet Gaming Disorder (IGD): From Psychological Features to Neuroanatomical Networks

**DOI:** 10.3390/jcm11041018

**Published:** 2022-02-16

**Authors:** Marilena Marraudino, Brigitta Bonaldo, Benedetto Vitiello, Giovanna C. Bergui, GianCarlo Panzica

**Affiliations:** 1 Neuroscience Institute Cavalieri Ottolenghi (NICO), Regione Gonzole, 10043 Orbassano, Italy; marilena.marraudino@unito.it (M.M.); brigitta.bonaldo@unito.it (B.B.); 2 Department of Neuroscience “Rita Levi-Montalcini”, University of Turin, Via Cherasco 15, 10126 Turin, Italy; gcbergui@gmail.com; 3 Child and Adolescent Neuropsychiatry, Department of Public Health and Pediatric Sciences, University of Turin, Via Cherasco 17, 10126 Turin, Italy; benedetto.vitiello@unito.it; 4 Fondazione Piccoli Cuori Joy, 12051 Alba, Italy

**Keywords:** IGD, GD, sexual dimorphism, psychological factors, neuroanatomical regions, gaming addiction

## Abstract

Internet gaming disorder (IGD) has been included in the 2013 *Diagnostic and Statistical Manual of Mental Disorders* (DSM-5) as a condition in need of further study, and gaming disorder was recognized by the World Health Organization as a mental disorder in the *International Classification of Disease* (ICD-11) of 2018. IGD has different characteristics in the two sexes and is more prevalent in males than females. However, even if the female gamer population is constantly growing, the majority of available studies analyzed only males, or the data were not analyzed by sex. To better elucidate sex differences in IGD, we selectively reviewed research publications that evaluated IGD separately for males and females collected in approximately one hundred publications over the past 20 years. The available data in this narrative review indicate that IGD is strongly dimorphic by sex for both its psychological features and the involvement of different brain areas. Impulsivity, low self-control, anxiety, emotion dysregulation, and depression are some of the psychological features associated with IGD that show a sex dimorphism. At the same time, IGD and its psychological alterations are strongly correlated to dimorphic functional characteristics in relevant brain areas, as evidenced by fMRI. More research is needed to better understand sex differences in IGD. Animal models could help to elucidate the neurological basis of this disorder.

## 1. Introduction

In the current highly digitalized world, gaming represents not only a recreational activity but also a potential threat when a person loses touch with reality, substituting gaming for social, occupational, or other recreational activities [1]. In 2013, the American Psychiatric Association (APA) included the internet gaming disorder (IGD) in the *Diagnostic and Statistical Manual of Mental Disorders, Fifth Edition* (DSM-5) [2]. In 2014, the World Health Organization (WHO) identified gaming disorder (GD) as a public health problem [2], and, in 2019, recognized it as a medical disorder to be included in the official list of health risks [3].

### 1.1. Diagnostic Criteria

Considering the paucity of clinical studies on IGD, one of the trickiest points to discuss is which are the best diagnostic criteria to apply in clinical practice [4]. In June 2018, WHO defined GD as characterized by specific diagnostic criteria that were listed in the 11th edition of the *International Classification of Diseases* (ICD-11) [6C51, Gaming disorder, ICD-11,4,5]. In particular, GD is defined in ICD-11 as a pattern of persistent or recurrent online (i.e., over the internet, reported as “predominantly online”, 6C51.0 ICD-11) or offline (‘digital gaming’ or ‘video-gaming’, reported as “predominantly offline”; 6C51.1 ICD-11) gaming behavior (WHO, 2018).

According to both DMS-5 [2] *American Psychiatric Association. Diagnostic and Statistical Manual of Mental Disorders* (DSM-5^®®^); American Psychiatric Publishing: Arlington, VA, USA, 2013] and ICD-11 [6C51, Gaming disorder, ICD-11], GD is characterized by a pattern of repetitive or persistent gaming behavior, which has to continue over a period of at least 12 months [4,5]. In Table 1, we recapitulate the diagnostic criteria included in DMS-5 (left column) and ICD-11 (right column).

To obtain a DSM-5 diagnosis of IGD, a patient must exhibit at least five out of nine symptoms, whereas, for an ICD-11 diagnosis of GD, all of the three criteria must be present [4,6]. The more stringent criteria for the IGD in the DSM-5 result in lower prevalence rates of IGD than GD. The threshold for IGD seems to be significantly higher than that for other addictions. For example, substance use disorders require only two out of eleven additional symptoms for diagnosis, whereas up to four out of nine symptoms are required for IGD [7].

### 1.2. Epidemiology

The prevalence of IGD worldwide seems to vary from country to country [8]. As described by Mihara and Higuchi [9], variable diagnostic criteria, assessment tools, and a different selection of the cohorts considerably limit the comparability of available studies, since a ‘gold standard’ for IGD has not been universally included yet [9]. In a very recent scoping review, to describe the prevalence of GD and IGD in the literature, the authors described 35 methods used to identify people with IGD, whereas no methods for GD were identified [10]. Depending on the selected study, although the most common methods of diagnosing IGD appear to be DSM-5 criteria and Internet Gaming Disorder Scale-Short Form (IGDS9-SF) [11], the prevalence of IGD using DSM-5 criteria can vary from 0.21% to 57.5% in the general population, from 3.20% to 91.00% in the population recruited for the IGD problem, and from 50.42% to 79.25% in samples with severe IGD symptoms [10]. Most of the studies were conducted among Asian countries’ populations, which seem to have a higher prevalence of IGD compared to non-Asian countries [8], but methodological differences between studies still make it difficult to properly compare studies [9]. Furthermore, for some geographical areas, such as Africa or South-East Asia, there are no studies in the literature that offer data on the prevalence of IGD.

Most studies have described a higher prevalence of IGD among young people than among the elderly, citing adolescence as “age at risk” [9,12,13]. However, several studies have identified, as new regular players, adults (between 18 and 29 years old) with specific characteristics, such as independence from parents and with fewer responsibilities than the elderly [14], being unmarried and unemployed, and with high rates of suicide attempts [15,16,17].

**Figure 1:**
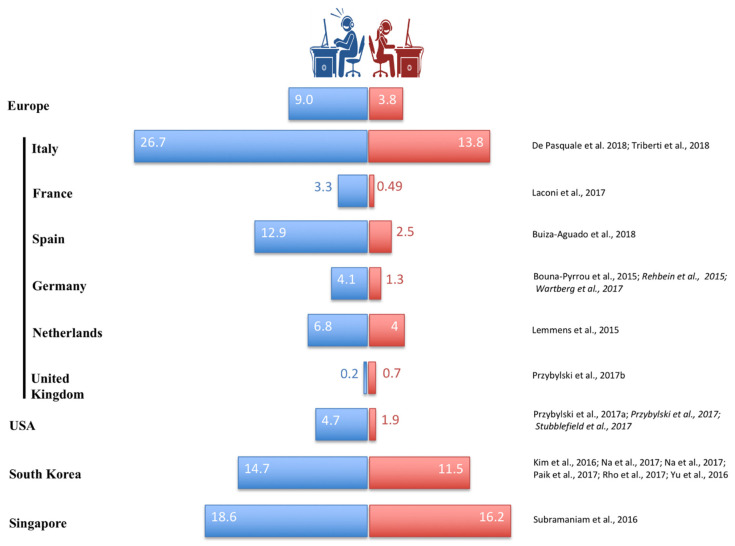
Internet gaming disorder prevalence in males and females. IGD prevalence (express as %) in a male and female general population obtained by the raw data provided in the study or by the average of these when the percentage of the prevalence of internet gaming addicted was extracted by sex in multiple studies. For the creation of this figure, only studies were used that analyzed males and females separately, in an age group between adolescence and adulthood.

The prevalence is usually higher in males than females (Figure 1) [8,9,18]. Darvesh et al. [10] reviewed 160 studies that used 35 different methods to diagnose IGD; these studies, conducted from general, clinical, and severe populations, highlight a prevalence range for males in the general population (0.21–57.50%) not undergoing treatment for IGD, and a strong male prevalence in severe cases (undergoing interventions, 50.42–79.25%), but these last data were based on only two studies. Regarding females, no studies were found for severe cases, but the prevalence in the general population seems to be from 0.25% to 26.09% in 21 analyzed studies. Furthermore, large numbers of psychopathologies have been displayed as comorbid to IGD [12], as we will discuss below. In particular, IGD-affected patients show symptoms linked to anxiety, depression, and attention deficit and hyperactivity disorder (attention deficit hyperactivity disorder, ADHD), and some studies also described association with social phobia, social anxiety, and obsessive-compulsive symptoms [12,19].

### 1.3. IGD and Cultural Factors

The cultural factors that influence different societal attitudes toward males and females and contribute to their accepted roles also have an impact on the use of video games and, therefore, on sexual dimorphism [20,21]. The prevalence and type of addictive behaviors vary across cultures [22]. The different cultural roles and expectations for males and females are reflected in the content of video games, which tend to be more designed for males. Since adolescence, female behavior tends to be more attracted towards social media, whereas males are more likely to engage in video games, possibly due to the stronger masculine attitude toward the competition intrinsic in these activities (Leonhardt et al., 2021). The relationship between culture and IGD is complex, influenced by many contextual social and economic factors, and likely mediated by the presence of other related psychopathological manifestations, such as social withdrawal [23].

The prevalence of behavioral addictions, such as IGD, is markedly greater in individualistic cultures, in which individuality, independence, and competitiveness can probably alter the social connection that leads to increased isolation, and therefore pathological dependencies to temper the way an individual feels [24]. If, then, the variable gender (understood as biological sex) is also inserted in the culture–IGD relationship, the question becomes even more complicated. If, in general, it appears that the risk of IGD is higher in adolescent males [25], when considering cultures that include hierarchy while maintaining autonomy and social independence (such as USA), then it appears that the gender difference and the risk of IGD are neutralized, as the tendencies for success and ranking will be less defined [26].

### 1.4. Historical Background

The interest in IGD is relatively recent, as previously anticipated: only in 2019 did the WHO recognize it as a medical disorder and include it in the official list of health risks. The very first work reported by Pubmed, in which Michael OReilly states that internet addiction could cause “the same kind of social problems as other established addictions”, is dated to 1996 [27]. The first works on the IGD do not differentiate the subjects studied by sex; most of the time, they focuses on males or do not specify. We have to wait until the end of the second decade of the 2000s to find papers in which a differentiated male population is recognized from a female one.

## 2. Objectives

Here, we present a narrative review of the current knowledge on sexual dimorphism in IGD, discussing in particular those studies that take into account both males and females, with particular attention to psychological and neuroanatomical alterations. Although ICD-11 classification is more recent and actually recognizes GD as a medical disorder, most of the available literature still uses the DMS-5 classification. Therefore, as of now, we will adopt the DSM-5 terminology IGD.

To develop this narrative review, we selectively reviewed research publications over the period 2001–2021 that had reported on IGD or GD for males and females. To carry this out, we selected all PubMed publications that included in the title, abstract, and MeSH terms the words IGD, gaming disorders, internet addiction, dimorphism, sex, or gender. Due to the focus on the sexual dimorphism, we have further selected within this group of articles those clearly reporting comparisons among sexes or at least clearly identifying the sex of the subjects. All papers reporting neuroanatomical alterations were also included. Approximately one hundred publications were selected as most relevant, in the authors’ view, to the review aims.

## 3. Sexual Dimorphism in IGD

In the IGD, there is a strong presence of sexual dimorphisms among problematic players, starting with the choice of video games: females prefer simulation games, whereas males prefer action games [28]. Studies show that males play mainly with shooting and role-playing games and are more attracted than females to violent and competitive elements without social interactions [29]. However, available studies on IGD have a strong limitation in common: the analyzed subjects are almost all males, and the data regarding females are very few. The possible explanation is that this disorder is more common in males than in females [25], partly because most video games were probably designed by males for males [30]. Furthermore, males have greater internet addiction during adolescence, when the time spent playing video games increases, whereas females develop greater use of the internet at an older age, where they can generate an addiction to social networks [31]. In general, it appears that females spend more time on the internet than males, but more for using social media (e.g., Email, Facebook, Twitter) than for gaming [32,33,34]. This may further explain why males have greater problems associated with multiplayer games than females [35].

A recent study of Lopez-Fernandez and colleagues [36] describes the female gamer profile and strongly underlines the importance of measures to diagnose IGD appropriately in both sexes. Indeed, over the past decade, the view on females has completely changed from non-players to moderate-strong players [37]. This is reflected in recent studies that have recruited participants from gaming groups who had a high prevalence of females. The female player stereotype of someone with a lower play competency and motivation compared to male players is receding [38]. The female population of gamers who enjoy playing the most popular online games is steadily growing [36].

## 4. Psychological Factors and Personality Traits Sexually Dimorphic in IGD

IGD includes individuals of heterogeneous sex, age, education, and culture. Several psychological factors, personality traits, and other psychological disorders appear to increase the risk of IGD. Impulsivity, poor self-control, anxiety, and the pursuit of desired appetitive goals are psychological features that have been found to be associated with IGD [39]. Female gamers seem to be more likely to develop poor mental health [34] and more psychiatric comorbidities than males [40].

### 4.1. Impulsivity and Poor Self-Control

Impulsivity and poor self-control are also found in other addiction behavior, such as gambling and substance abuse [41]. Several studies show that male gamers with higher levels of impulsivity are more at risk of developing IGD [42,43,44]. In particular, impulsivity appears to be related to addiction to networking sites or the use of smartphones, whereas low self-control and anxiety are factors that increase the chances of general internet use and video game addiction [45]. Compared to non-problematic gamers, problematic gamers showed signs of emotional dysregulation, such as a difficulty in describing feelings, the tendency of individuals to focus their attention on the outside, and higher scores on an alexithymia subscale, which expresses an emotional unawareness, lack of social attachment, and poor interpersonal relationship [28]. As they grow up, these young adults develop an adaptation by finding in games a way to alleviate the emotional dysregulation associated with alexithymia, without being able to find an alternative way to express their emotions [46]. A recent report by Bonnaire and Baptista [28] shows that this aspect of IGD is dimorphic: alexithymia is a risk factor for IGD only in males, confirming previous data supporting sex differences in the emotional expression and experience [47]. Male gamers have more than doubled the risk of having IGD if they are also alexithymic, young, and have high anxiety and depression scores [28]. The authors hypothesize that alexithymia may be a constant personality trait in male players, presenting a risk factor for IGD, whereas, in female gamers, it is a consequence of psychological distress, depression being the real female risk factor for IGD [28]. However, in this study of 429 participants (with a mean age of 20.7 years), only 28.7% were females. Therefore, if alexithymic females were not identified by the authors, this could depend on the sample size of the participants. It will take several reruns to obtain more convincing data.

Closely associated with impulsivity is hyperactivity (ADHD), one of the disorders that, more than others, has been shown to increase the risk of a player experiencing symptoms of IGD [43,48,49,50]. ADHD is a developmental condition defined by a “persistent pattern of inattention and/or hyperactivity-impulsivity that interferes with functioning or development” (APA, 2013). ADHD has a male prevalence: affected males have more externalized symptoms of hyperactivity and impulsivity, whereas inattention prevails in females. Interestingly, both hyperactivity–impulsivity and IGD could arise from a deficit in self-control, and it seems that male players with attention deficit and hyperactivity–impulsivity have a higher IGD risk [51]. As well described by Stavropoulos et al. [51], IGD may be bi-directionally associated with ADHD. Whereas some reports have suggested that ADHD features are predictors of IGD, others have shown that IGD behaviors emphasize ADHD symptoms [50,52], and that excessive gambling could be a way of escaping reality in people with ADHD [53]. An interesting study by Yen et al. [54] found that attention deficit and impulsivity were the two most commonly associated symptoms linking ADHD and IGD in college students, and that this association was stronger in females than males.

### 4.2. Hostility and Social Phobia

Hostility [55] and social phobia [56] are possible risk factors of IGD. A study reported that their presence was common among individuals with gaming disorder [57], and another study in female players suggested that hostility and social phobia could explain an increased irritability and low propensity to share thoughts and time with other female players [36]. The study of Ko et al. [58] reports that social phobia, as well as depression, are predictors of subsequent internet addiction among females, but not males. Furthermore, it appears that loneliness may influence the rise of instant messaging [59], on which, females are more dependent. This sex difference was not found in adolescents [34]; the authors explained this incompatibility with the use of a questionnaire that was too broad to detect the consequences of poor mental health.

### 4.3. Depression

Another important psychological factor associated with IGD is depression [60], which affects females more frequently than males [60,61,62]. Several studies have found an association between the presence of depression in adulthood and a history of emotional trauma in childhood [63,64]. In Korean adults, IGD, as compared to other mental disorders, such as alcohol addiction and anxiety, showed a strong association with depression and other related negative emotions, such as sadness, nervousness, and anger [15]. In this same study, although the authors report a percentage of 32.4% of the IGD females analyzed (out of a sample of 1401 adult individuals), the analysis of the multivariate logistic regression of psychiatric comorbidities is analyzed with adjustments (for age, sex, years of education, marital status, and all variables above), but without distinguishing between internet gaming addicted and non-addicted [15]. Furthermore, a recent study reported that depression is not only a risk factor for IGD, but also that individuals who have become addicted to video games and experienced emotional abuse and/or abandonment as children have a high rate of depression [64]; however, the group of females was too small to evaluate the effects in terms of sexual differences. These data support the idea that IGD may be a “maladaptive coping mechanism when dealing with emotional disorders and/or traumatic life events” [64].

### 4.4. Aggressive Behavior

Another interesting sex-dependent psychological manifestation in IGD players is aggressive behavior [65]. Overall, IGD teenagers demonstrated a close association between gambling addiction and aggression [66], with high hostility scores, involvement in fights, and arguments with peers and teachers within the previous year [67]. The use of violent games seems to arouse aggressive emotions [68], and many popular video games have different levels of graphic violence [69]. IGD males seem to exhibit more aggressive feelings than females; in fact, violent video games are associated with aggressive behavior and offline delinquency and, unlike females, males present a more hostile view of the world [70]. For this reason, many violent video games promote sexual objectification through the use of male avatars. It appears that female players, even if they change sex in the game, do not respond to the violence with aggressive behavior, but perceive violence to be part of the games they play [36].

## 5. Sexual Dimorphism in the Brain of IGD Gamers

Several functional magnetic resonance imaging (fMRI) studies have analyzed the presumed brain regions involved in IGD. However, they have limitations mainly related to the subjects that were studied, because they were almost all males or, even when both sexes were considered, the analyses usually did not consider sex [71,72,73]. The studies that have evaluated males and females separately, taking into account a possible sexual dimorphism in the different brain areas of interest in IGD, are very few [74,75,76]. Neuroimaging studies performed in both sexes showed that changes in resting-state activity were associated with IGD in brain regions responsible for attention and control, decision-making, and sensory-motor coordination (e.g., prefrontal cortex, PFC) [77], parietal cortex, posterior cingulate cortex (PCC), and cortical-ventral striatum circuitry [78,79] (reported in Table 2).

### 5.1. The Prefrontal Region

The frontal lobe plays a key role in future planning, including self-management and decision-making. PFC structural and functional abnormalities have been, in fact, related to a high impulsivity, a trait that may contribute to the altered inhibitory control associated with IGD [77]. Imaging studies have characterized how both the structures and functions of the frontal lobe are altered in association with impaired inhibitory control in individuals with gaming addiction [80,90]. Dieter et al., in an fMRI study, reported a correlation between the functional alterations in PFC and a high impulsivity in IGD [91], which is also confirmed by the work of Han and colleagues [92]. The authors, using resting-state fMRI, demonstrated that lower functional connectivity between the left medial orbitofrontal cortex and the putamen in a group of gaming addicts was significantly correlated with the Barratt Impulsiveness Scale-11 (BIS-11, a scale that evaluates the behavioral inhibition function of IGD), thus associating alterations of prefrontal–striatal circuits with impulsive behavior [92].

The prefrontal region includes the dorsolateral prefrontal cortex (DLPFC) and is connected to the striatum; many studies correlate these areas with the duration of gaming, cognitive impairments, and the severity of IGD [85,93]. In particular, in young adult males, Li and colleagues, combining regional gray matter volume (rGMV) analysis and resting-state functional connectivity, showed a relationship between rGMV in right DLPFC and the severity of IGD and cognitive inhibitory control [72]. Additionally, another study in males by Choi et al. reports the association between lower gray matter density in the left DLPFC and more severe symptoms in internet gaming groups, such as more depression and impulsivity, and more time spent in gaming [73]. The authors hypothesize the left DLPFC as a potential biomarker for the depression modality present in IGD, because addicted individuals with an altered left DLPFC (the region more responsive to positive emotions than the right DLPFC) may not be able to respond to pleasant stimuli [73].

Only two studies analyzed the sexual dimorphism in DLPFC, associating this region with the desire to play. Dong and colleagues, using fMRI, compared the pre- and post-game phases, showing less involvement of the left DLPFC in all players, and mostly in females. Furthermore, addicted females had higher activation in the caudate than females engaged in recreational play (RGU) [94]. These results suggest that females are less prone to developing an addiction to internet game playing than males, but, when this does happen, addicted females may have an impairment in their executive control and an increased desire for gaming, which makes it harder to stop gaming [94]. In a more recent study, Dong et al. [76] analyzed functional connectivity and observed a sexual difference. In fact, during the game, and only in males, the activity between the DLPFC and superior frontal gyrus decreases, but increases between the striatum and thalamus., whereas, during the mandatory break, the functional connectivity seems to be relevant for both sexes, and, in particular, for addicted females. This could explain once again why males develop IGD more often than females, and why it can be difficult for addicted people to quit gaming [76].

### 5.2. Posterior Cingulate Cortex (PCC)

Together with the medial prefrontal cortex (mPFC), the posterior cingulate cortex (PCC) is a key component of the default mode network (DMN). Indeed, PCC is extremely important in connecting the DMN to task-related networks [95,96]. Several studies show that, in IGD, there is a decreased functional connectivity (rsFC) between the DMN-related regions, including the PCC, during the resting state [79,97]. A recent study [75] described that IGD patients show a reduced coupling from the mPFC to the PCC compared to RGU and that there is an increase in the directional inhibition of the left IPL (inferior parietal lobule)-mPFC-PCC neural pathway. Furthermore, higher fractional anisotropy, both in the PCC and in the thalamus, has been found to be associated with a greater severity of IGD [76]. These results confirm the notion that the PCC is a fundamentally driven hub that is necessary to integrate information and to project it to major brain structures, and that the dysregulation of the pathway from the mPFC to the PCC could be a useful biomarker to distinguish IGD patients from healthy individuals [75]. The decrease in rsFC between the PCC and supplementary motor area has already been proposed as neurological evidence of the efficacy of behavioral interventions for IGD [98]. Indeed, the PCC is indispensable for the coupling of executive control and the coordination of the endogenous activity of the brain during the resting state, which are necessary for attention and self-monitoring [95,96]. 

According to Wang et al. [75], in IGD, the cognitive function is impaired only in affected males. In these subjects, the right PCC has a lower regional homogeneity compared to RGU: this is linked to the decline of regional synchronization in the PCC during the resting-state and it is negatively correlated with the Internet Addiction Test (IAT) score. Interestingly, Hoeft et al. [74] have previously reported that, in healthy subjects, males show greater activation and functional connectivity in the limbic system during play than females: this could be associated with a weakened function of risk assessment of internet gaming or with a compensatory mechanism of the weakened risk assessment function. The decrease in PCC’s regional homogeneity itself, which is higher in males [75], may be involved in the immersion of the internet gaming, meaning that males with IGD are more likely to be immersed [95], and may be linked to gaming motivation: stronger participation motivation is highlighted in the questionnaire on motivation in males with IGD compared to females, whereas females show higher entertainment motivation [75]. Therefore, motivation seems to be a key factor to consider in improving the studying of sex-related differences in IGD. Moreover, the male mesocorticolimbic system is more activated during game playing in males, which displays a higher motivational state [74]. Instead, as hypothesized by Wang and colleagues, females seem to be more vulnerable to IGD than males [89]. Using structural MRI, the authors found that, in females with IGD, there is an increased cortical thickness in the right PCC, with a negative correlation with cravings and self-reported ITA scores [89].

### 5.3. Brain Regions Involved in Visual Processing and Cognitive Control

In addition to what has been previously described, subjects with IGD show higher activation in the inferior parietal lobule (IPL) and in the middle occipital gyrus (MOG) [99], which are areas that, together with DLPFC, are involved in visual processing and cognitive control during gaming [98]. Furthermore, a decrease in the volume of gray matter in the inferior temporal gyri, the middle occipital gyrus (MOG), and the inferior occipital gyrus was also observed [100]. Unfortunately, these studies are of limited value, as they were mainly performed in male subjects, while, recently, Wang et al. [75] have shown that audiovisual functions are affected differently by IGD in males and females. The left middle occipital gyrus (lMOG) and the right middle temporal gyrus (rMTG) in IGD-affected males display a higher regional homogeneity (ReHo) than in RGU males, whereas, in IGD-affected females, the ReHo is lower in these regions than in the RGU females [75]. The lMOG, located in the occipital lobe, is fundamental for the processing of the visual and visual–spatial information, color resolution, and motion perception [101], whereas the rMTG, located in the lateral of the temporal lobe, is fundamental for the processing of auditory information [102,103,104]. Interestingly, sex differences in ReHo are present not only in the IGD groups but also between males and females of the RGU groups [75]. This observation is also supported by previous studies, which demonstrated that (I) the audiovisual area is sex-different [105], (II) different strategies for managing auditory information can be highlighted thanks to sex-different local BOLD signal patterns in temporal-lobe regions [106], and (III) males and females show regional-specific differences in spontaneous brain activity in areas involved in the primary visual network [107]. 

Sex-related differences in IGD patients have also been found in the right postcentral gyrus (PG), which, as the primary somatosensory cortex, is involved in the management of tactile information [108]. Although some studies have already been published [109], the role of PG in the addiction pathway is still unclear. One hypothesis is that the players’ response to game information may involve keystrokes, which could be linked to changes in the right PG and, therefore, this region may also represent an interesting target for studies of sex differences in the IGD [110].

### 5.4. Mesocorticolimbic Reward System

Another important target for IGD is the mesocorticolimbic reward system, including the nucleus accumbens (NAc) and the orbitofrontal cortex (OFC), which are part of the brain dopaminergic system. Few works have demonstrated the important role of dopamine, but only in male video game players. In 1998, Koepp et al. [71], using positron emission tomography (PET), demonstrated an increase in the release and binding of dopamine in the ventral striatum in video game players. In 2006, using near-infrared spectroscopy (NIRS), another study demonstrated a decrease in activity in the dorsal prefrontal cortex [111], which modulates dopamine release in the limbic system [112]. In this scenario, dopamine plays an important role as a processor of salient stimuli (e.g., gaming pictures) [113], because salient information acts on dopamine neurons, decreasing the sensitivity of natural stimuli and obtaining a dysfunctional reward [114,115], which may be associated with the depression mode present in individuals with IGD [73]. The first fMRI study of computer video game players examined the sexual dimorphism in neural systems related to reward and addiction and showed that sexual differences existed in the NAc and OFC [74]. The authors demonstrated that, during an implicit space-infringement task, males show a higher activation and functional connectivity than females, speculating that the overlap with neural processes underlying addiction may explicate the greater male inclination to play video games in a repetitive manner [74].

## 6. Correlation of Neural Sexual Dimorphisms between IGD and Substance Abuse

There are sex difference in substance abuse, such as alcohol, cocaine, nicotine, and smoking, as well as in IGD. Females are more inclined to develop a state of dependence, anxiety, and depression following cocaine and gambling addiction [116]. In general, many studies reported a strong correlation between alterations in the mPFC and substance abuse [72,117,118]. An imaging study showed that cocaine-dependent females consistently presented higher PFC activity than males [119]. Problems in making good decisions characterize addictions, including IGD, in which individuals have deficits in impulse control. The neuronal network that involves the prefrontal–striatal circuits (which includes OFC–, anterior cingulate cortex (ACC)–, inferior frontal gyrus (IFG)–, and DLPFC–striatal circuits) is an important target in these pathological conditions [113]. Jin et al. [120], using voxel-based morphometric and functional connectivity, showed that the regions of prefrontal–striatal circuits are effectively altered in IGD. In this study, participants were young adults with IGD, but with a low representation of females, as indicated by a 16/9 male/female ratio.

Other brain regions relevant to IGD and other addictions showed a strong sexual dimorphism: the superior frontal gyrus (SFG) and PCC were found to be more active in tobacco-addicted females than in males [121], and, again, in methamphetamine (METH)-addicted females, the right SFG was smaller and NAc larger than in addicted males, which, according to the authors, is suggestive of an estrogen-mediated neuroprotective glial response [122]. In the same study, Kogachi and colleagues pointed out that the larger superior frontal cortex (associated with a greater cognitive impulsivity) in male METH users (possibly caused by a decreased dendritic pruning during adolescence) could contribute to their impulsive behavior and drug addiction [122]. This was reported in IGD-addicted male adolescents who have a highly impulsive behavior, connecting, again, sex differences with IGD and substance abuse. Further, the association between different morphology and sex addiction also involves the insula, another important region of the limbic system, which controls anxiety, avoidance learning, and drug cravings [123]. The authors, using the morphometric analysis correlated to the decision-making performance, show that, in tobacco- and alcohol-dependent females, the volume of the insula is smaller than in males. 

## 7. Limitations in IGD Human Studies

The studies reviewed here have several limitations, mostly due to the characteristics of the clinical samples used in the available studies. As often pointed out in this review, one of the problems is a poor consideration of the sex of the analyzed participants. Many studies recruited only male participants, or, when males and females are recruited, the data were then not analyzed separately by sex. It will therefore be necessary in future studies to increase the number of female participants to confirm the current results and to better understand the data on sexual dimorphism in IGD. Another important aspect is the age of the participants. In general, studies have been limited to adolescents or college students, but many observations show that IGD can also occur in young adults or adults. Furthermore, age is an important factor that has a different effect on the development of IGD in both sexes: males develop gaming addiction during adolescence, whereas females develop it later in life. Thirdly, in these studies, the different potential variables analyzed, such as the demographic distribution, culture, IQ, economic status, and family status, are very few, but they could have a great impact on IGD (as reported in Table 1, the large majority of studies were performed in China). 

Another limitation relates to the recruitment method: many data come from online sources interested in the virtual game culture, where participants fill in the questionnaires administered online and independently answer all of the questions presented, including those relating to their sex or psychological state.

A major limitation in the currently available IGD studies is the concept of a ‘control group’. In many cases, the individuals used as controls are game users for recreational purposes (RGU), which limits the generalizability of the data and does not explain well the possible psychological or neuroanatomical changes reported in users addicted to gaming. To get around this problem, a third group composed exclusively of individuals of both sexes with no internet gaming experience should be added to the RGU group. Another limitation observed in some studies is the small size of the analyzed sample, especially in the rare cases where both sexes are present. This may increase the risk of false negatives, but also does not emphasize the real differences that may exist in male and female IGD.

Particularly, in many studies that assessed the psychological characteristics, only a single measure was used or a single psychological factor was evaluated as a predictor of IGD. This is an important limitation given the considerable heterogeneity of game users with respect to psychological features. Moreover, many individuals may present pathological alterations even before developing the IGD, while others may manifest them as a consequence of IGD. In addition, many other variables can be associated to psychological factors, such as the type of game played, the age, and the sex of the player. More comprehensive studies are needed in the future to identify which psychological factors could be used as predictors of IGD and the possible differential role of sex. Finally, the most important goal in human studies is to identify unique and common diagnostic criteria for IGD by studying the various factors that we have previously reported. Today, nine criteria are used for the clinical diagnosis of IGD according to the DSM-5 definition, but, of these criteria, seven are common with gambling disorder and five with substance abuse disorder [124]. The identification of specific criteria for IGD could help to identify the disorder and, also through the use of scales, such as the Gaming Addiction Scale (GAS), could allow for its prevalence to be better estimated. 

## 8. Beyond Human Study: The Animal Model for the Study of IGD

In this initial phase of investigation into the psychological and neuroanatomical correlates of the IGD, an animal model could be a useful support to better understand the circuits involved and to identify possible approaches to diagnosing, preventing, and treating. An animal model would obviate and address some of the limitations of human research that were described in the previous paragraph. It is possible to hypothesize an animal model of IGD starting from studies that have proposed animal models for other addictions (gambling disorder), or by the application of technologies, such as touch-screens, that have been used in research on memory and learning. Gambling disorder is another addictive disorder that shares many psychological and neuroanatomical alterations with IGD. The first animal model for slot machine gambling was designed by Weatherly and Derenne (2007) and further improved by Peters et al. to include near-winning trials [125]: the apparatus with a ‘spin’ level has light stimuli and a food dispenser used as a reinforcement. Following Pavlovian conditioned reinforcement, food is used in the animal model to reward winning trials as an analog of slot machine credits for humans. Instead of food, another animal model for gambling disorder uses intracranial self-stimulation as a reward [126,127], thus eliminating potential feeding-related confounders. For the study of gambling behavior, these animal models have been fundamental to advance the knowledge of the neural and psychological basis of the disorder, but also to study possible pharmacologic strategies. 

Similarly, an animal model for IGD brain examination and analysis could be important for understanding the sexual dimorphism and the presumptive role of hormones in this disorder, and the possible involvement of neurotransmitters and neuropeptides, such as dopamine, oxytocin, or vasopressin, and their receptors. Moreover, an animal model could provide a solid basis for the development of future studies on possible familiar (as maternal care) and environmental factors that may interfere with IGD, but also possible drug treatments. Furthermore, an interesting new apparatus that features a touchscreen platform is only currently being used to study learning and working memory through many cognitive tests that are very similar to touchscreen devices used daily by humans [128]. As underlined by the authors, the use of this apparatus develops a loss of stress in animals and has great translational potential, especially in animal models of psychiatric and neurodegenerative diseases. Another possible application of this methodology with new setups adapted to the IGD, together with the knowledge of animal models for addictive disorders, could be a good starting point for developing an animal model IGD.

## 9. Conclusions

Internet gaming disorder (IGD) is a clinically identifiable disorder, but the risk of developing this disorder is not easy to understand due to the lack of clear predictive signals and the presence of many potentially contributing factors. The claim that males are at greater risk for IGD than females is not supported by clear-cut evidence. The purpose of the review was to highlight sexual differences in the IGD and psychological and behavioral, as well as neurobiological, differences between IGD patients and non-IGD subjects. Analysis of the literature has evidenced some important limitations in the existing literature. Very few studies analyzed the data by sex or, when they did so, used small samples. Most of the studies examined psychological factors, whereas studies of the involved circuits with the fMRI technique are still few. The examined cohorts come largely from East Asia populations or undefined populations (see Table 2), thus limiting generalization, since cultural and social differences probably play an important role in the development of this disorder.

Despite such limitations, the data currently available from research in humans confirm the existence of sex differences in the attitude towards internet games, the development of the IGD, and the age in which these differences first appear. There are also sex differences in the circuits that control attention, decisions, and sensory-motor coordination, in both their structure and the differentiated responses.

On the whole, considering both the evidence and the limitations, it seems of extreme interest to develop animal models that can complement human studies, as already implemented for addiction studies. In our opinion, a significant effort must be made to develop these models to better clarify the neural circuits and sex differences linked to IGD. The development of animal models will also be important to test different environmental situations and putative pharmacological interventions.

## Figures and Tables

**Table 1:** Diagnostic criteria for DSM-5 and ICD-11. *Diagnostic and Statistical Manual of Mental Disorders* (DSM-5) included in 2013 internet gaming disorder (IGD) as a condition for further study (left column), and in 2018, the World Health Organization included gaming disorder (GD) as a mental disorder in the *International Classification of Disease* (ICD-11) (right column).

DMS-5	ICD-11
1. Preoccupation	1. Impaired control Impairment—Personal Impairment—Social Impairment—Education Impairment—Work Impairment—Financial
2. Withdrawal	
3. Tolerance	
4. Unsuccessful attempts	
5. Loss of interests	
6. Continued use	
7. Deception	
8. Escape	2. Increasing priority
9 Jeopardized life	3. Continuation

**Table 2:** Neuroanatomical regions affected in male and female IGD. Studies analyzed internet gaming disorder (IGD)-affected individuals, both males and females, and controls. For each study, analyzed neuroanatomical regions, size of the samples, male-to-female ratio, presence of sex-by-group analysis, race/geographical origins of the participants, mean (±standard deviation) of the participants, employed methods, and observed effects are reported. Internet-gaming-disorder-affected individuals (IGD), controls (Ctrl), gray matter (GM), white matter (WM), functional magnetic resonance imaging (fMRI), functional connectivity (FC), regional homogeneity (ReHo), voxel-based morphometry analysis (VBMA), tract-based spatial statistics (TBSS), fractional anisotropy (FA), diffusional kurtosis imaging (DKI), voxel-mirrored homotopic connectivity (VMHC), right (r), left (l), bilateral (b), inferior (i), superior (s), middle (M), median (m), cingulate gyrus (CG), para-hippocampus (pHIPP), frontal lobe (FL), frontal gyrus (FG), post-central gyrus (PCG), occipital gyrus (OG), temporal gyrus (TG), orbitofrontal cortex (OFC), supplementary motor area (SMA), external capsule (EC), parietal lobule (PL), parietal gyrus (PG), lateral lingual gyrus (LLG), paracentral lobule (PCL), cingulate cortex (CC), fusiform gyrus (FFG), posterior cingulate cortex (PCC), anterior cingulate cortex (ACC), dorsal lateral prefrontal cortex (DLPFC), rostral middle frontal gyrus (RMFG), supramarginal gyrus (SMG).

Neuro Anatomical Region	Sample (IGD/ Ctrl)	M/F	Sex-by-Group-Analysis	Race/ Geographical Regions	Age	Methods	Observed Effects	References
Cerebellum, brainstem, rCG, bpHIPP, rFL, lSFG, left pre-cuneus, fPCG, rMOG, rITG, lSTG, MTG	19/19	11/8 IGD 11/8 Ctrl	No	Chinese	n.c	fMRI to measure ReHo	↑ ReHo	[80]
rOFCR, bilateral insula, rSMA, right genu of corpus callosum, bFL, rEC	17/17	4/13 IGD 2/15 Ctrl	No	Chinese	16.25 ± 3.0 15.54 ± 3.2	MRI (VBMA and TBSS) to measure GM density and WM density changes	GM atrophy in the rOFC, bilateral insula, and rSMA ↓ FA in WM of right genu of corpus callosum, bFL rEC	[81]
Cerebellum, TG, iPL, iPG	17 /24	13/4 IGD 16/8 Ctrl	No	n.c.	16.94 ± 2.7 15.87 ± 2.7	fMRI to detect FC	↑ FC in the bilateral cerebellum posterior lobe and mTG ↓ FC in the biPL and riTG	[82]
Right anterolateral cerebellum, risTG, rSMA, MOG, right pre-cuneus, PCG, riFG, lLLG, lPCL, laCC, mCC, bFFG, insula, PCC, thalamus	18/21	15/3 IGD 18/3 Ctrl	No	n.c.	20.5 ± 3.5 21.95 ± 2.4	MRI followed by DKI in the detection of GM diffusion	↓ GM diffusion in all analyzed regions	[83]
Cerebellum, ACC, SMA, sPL, precuneus, insula, DLPFC, FG	28/28	18/10 IGD 20/8 Ctrl	No	n.c.	18.8 ± 1.3 19.3 ± 2.6	fMRI to measure GM volume, FC and VMHC method	↓ GM volume of the bilateral ACC, pre-cuneus, SMA, SPL, left DLPFC, left insula, and bilateral cerebellum ↓ VMHC between the left and right sFG (orbital part), iFG (orbital part), mFG and sFG	[84]
Striatal nuclei (caudate, putamen, and nucleus accumbens)	27/30	23/4 IGD 22/8 Ctrl	No	n.c.	17.9 ± 0.9 18.3 ± 1.6	fMRI to detect FC and to measure volumes	↑ volumes caudate and nucleus accumbens	[85]
ACC, DLPFC	28/25	17/11 IGD 16/9 Ctrl	No	n.c.	19.3 ± 2.1 19.7 ± 3.8	fMRI to detect FC	↓ FA in the salience network, right central executive network tracts, and between-network (the ACC-right DLPFC tracts)	[86]
sPL, precuneus, CG, sTG, brainstem	19/19	11/8 IGD 11/8 Ctrl	No	n.c.	21.4 ± 1.0 20.8 ± 1.1	Task-state in fMRI	↑ activation in the right SPL, right insular lobe, right precuneus, rCG, right STG, and left brainstem.	[87]
PCC, mPFC, iPL	64/63	36/28 IGD 41/22 Ctrl	No	n.c.	22.577 ± 2.2 23.085 ± 2.5	Resting-state fMRI to detect connectivity	↓ interactions between the left IPL-mPFC-PCC (↑inhibition)	[75]
rPCC, lMOG, rMTG, and rPCG	46/58	23/23 IGD 29/29 Ctrl	Yes	n.c	23.0 ± 1.1 22.7 ± 1.4	fMRI to measure ReHo	♂: ↓ ReHo in rPCC, ↑ ReHo in lMOG and MTG ♀: ↓ ReHo in lMOG and MTG	[88]
DLPFC, striatum, thalamus and insula	54/65	29/25 IGD 34/31 Ctrl	Yes	n.c	21.14 ± 2.4 21.17 ± 2.1	fMRI to evaluate FC	♂: ↓ FC between DLPFC and sFG ↑FC between striatum and thalamus/insula ♀: ↑FC between striatum and thalamus/insula	[76]
bRMFG, sFG, lSMG, rPCC, rsPL	62/71	29/33 IGD 37/34 Ctrl	Yes	n.c.	21.1 ± 1.4 20.7 ± 1.8	Structural MRI to evaluate cortical thickness	♂: ↑ bRMFG, sFG, lSMG ↓ rPCC ♀: ↓ bRMFG, sFG, lSMG ↑ rPCC	[89]
